# Vision-Based Suture Tensile Force Estimation in Robotic Surgery

**DOI:** 10.3390/s21010110

**Published:** 2020-12-26

**Authors:** Won-Jo Jung, Kyung-Soo Kwak, Soo-Chul Lim

**Affiliations:** Department of Mechanical, Robotics and Energy Engineering, Dongguk University, 30, Pildong-ro 1gil, Jung-gu, Seoul 04620, Korea; iamdnjswh@dongguk.edu (W.-J.J.); akks22@dongguk.edu (K.-S.K.)

**Keywords:** force estimation, interaction force, neural networks, machine learning, minimally invasive surgery, suture tensile force

## Abstract

Compared to laparoscopy, robotics-assisted minimally invasive surgery has the problem of an absence of force feedback, which is important to prevent a breakage of the suture. To overcome this problem, surgeons infer the suture force from their proprioception and 2D image by comparing them to the training experience. Based on this idea, a deep-learning-based method using a single image and robot position to estimate the tensile force of the sutures without a force sensor is proposed. A neural network structure with a modified Inception Resnet-V2 and Long Short Term Memory (LSTM) networks is used to estimate the suture pulling force. The feasibility of proposed network is verified using the generated DB, recording the interaction under the condition of two different artificial skins and two different situations (in vivo and in vitro) at 13 viewing angles of the images by changing the tool positions collected from the master-slave robotic system. From the evaluation conducted to show the feasibility of the interaction force estimation, the proposed learning models successfully estimated the tensile force at 10 unseen viewing angles during training.

## 1. Introduction

Conventional open surgery requires a large incision, but minimally invasive surgery (MIS) only requires a small incision. MIS is popular because it minimizes the recovery time and cost as well as having cosmetic benefits. Robotics-assisted minimally invasive surgery (RAMIS) solves hand tremor and eye-hand coordination problems that exist during MIS. A surgical robot provides dexterous movements combined with a high vision quality [1]. However, applying force feedback remains a problem because there is no proper way to measure the interaction forces [2,3]. One of the major issues caused by this problem is that the sutures break owing to excessive traction. The problem of a suture breakage from excessive pulling complicates the surgical process, increases costs, and adds risk by requiring additional corrective measures if detected intraoperatively [4]. In addition, if weakened sutures break inside the patient postoperatively, the patient may suffer a hemorrhage from vascular anastomosis [5] or peritonitis due owing to a bowel anastomosis disruption [6]. For these reasons, it is necessary for the surgeon to estimate the tensile force of the suture and ensure that the sutures are applied correctly.

There are two main methods to overcome the absence of force feedback: direct force sensing and sensorless force estimation [7]. In direct force sensing, the force is measured at or near the point where the surgical tool and organs interact. Abiri et al. [8] developed a device using a piezoresistive sensor that can be attached to the tip of a surgical tool. In addition, Reiley et al. [9] used a strain gauge attached along the shaft of a surgical tool to measure the interaction force. This provides the most intuitive solution, but comes with numerous constraints such as cost, biocompatibility, sterilization, and miniaturization. For example, the most commonly used method for sterilization is an autoclave, which destroys electronic devices owing to high temperature, pressure, and humidity [10]. Devices [11,12] using fiber Bragg gratings (FBGs) to measure the interaction forces can endure high-temperature sterilization, although the optical fiber used by FBG is less durable and has mechanical limits under complex motions.

By contrast, sensorless force estimation methods predict the force through the already available resources without the use of additional electronic devices. Owing to the aforementioned problems with direct force sensing, there is more potential for the sensorless force estimation to be used in RAMIS [7]. A sensorless force estimation can be divided into control and vision-based approaches. Control-based approaches use observers, models of the surgical tool, and available information from motor devices (e.g., angular position/velocity, current consumption, and torque) to estimate the interaction force [7]. From this perspective, Zhao et al. [13] developed a method using modeling information from the surgical tools and the current of the motors to predict the force. To demonstrate the accuracy of the system, they then created a force-feedback approach using their method for classifying the differences in porcine liver stiffness. Wang et al. [14] measured the external force using a cable-tension disturbance observer for a surgical robot end-effector. However, using these control-based methods, it is difficult to obtain accurate modeling information of surgical tools or robot manipulators. It is therefore difficult to create an accurate interaction force prediction method that relies on this modeling and motor information. Because the motor is behind the trocar and is influenced by external factors, it is difficult to accurately predict the interaction forces between the forceps and organ from the available motor information. Vision-based approaches usually predict the force from visual information. These predictions are refined using surgical tool information such as the tool-tip trajectory, velocity, and grasper status. A number of vision-based approaches have been developed to estimate forces based on 2D or 3D deformation [7,15]. Studies for predicting and measuring the shear force on an image are also being conducted [16,17]. Such approaches first determine the amount of deformation caused by certain applied forces in advance. The relationship between deformation and force is then calculated using the mechanical properties. Kennedy et al. [18] studied the measurement of forces on a rubber membrane. The deformation was calculated by tracking the nodal displacements. A finite element method was used to determine the forces based on these deformations.

**Figure 1:**
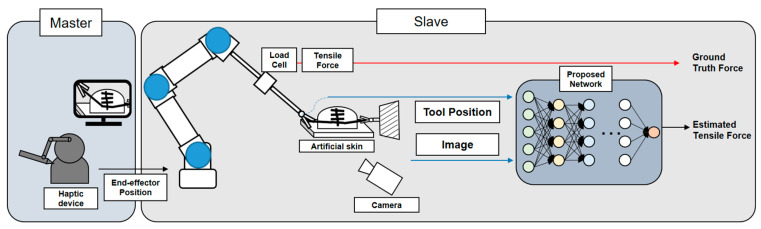
Robotic surgery experiment system for vision-based suture tensile force estimation. Simplified procedure of the proposed method and its experiments. An operator controlled the robot with visual information by sending the position of the end-effector. On the tool, there is a load cell that measures the ground truth of the tensile force. The force was estimated from the image and tool positions using the proposed network.

Figure 1 shows proposed a novel suture tensile force estimation method that is constructed to extract spatiotemporal features from both the image and tool positions. Using a deep-learning model, this method is designed to mimic the tension prediction using the sense of proprioception and vision. To validate the general performance of the proposed method, a robotic surgery system that interacts with skin and sutures is used. A database of changing soft objects, camera views, and amounts of target tensile force was collected. The database was used as the training and test sets of the model.

## 2. Related Works

### 2.1. Vision-Based Force Estimation

Vision-based force estimation uses the fact that soft bodies made of biological (e.g., tissue) or artificial (e.g., silicone) materials deform when forces are applied to them. In vision-based force estimation, this deformation of the soft body when force is applied is captured by a camera. This information is then used to predict the actual force being applied to the object [19,20]. Previous vision-based force estimation methods used various algorithms to create models for deformation and found relationships between the deformation and forces. Karimirad et al. [21] proposed a method to predict forces during the microinjection of the zebra embryos. This study utilized active contours and a conic fitting algorithm to model the contour deformation of the cell. A fully connected neural network (FC) learn the mechanical relationship between the deformation and the force. In a method proposed by Kim et al. [22], soft tissue surface deformation was calculated from a stereo endoscope image. This information was then applied to a surface mesh (based on spring-damper models) to estimate the force. This image deformation information along with information on the stiffness of the organ was used to determine the interaction force between the tissue/organ and tools for robot-assisted surgery [23] and neurosurgery [24]. Naeini et al. [25] proposed measuring the contact force with a neuromorphic camera(dynamic vision sensor) using Long Short Term Memory (LSTM) networks. However, recent vision-based force estimation approaches have applied deep learning to predict the interaction forces from images using both spatial and temporal information without the need for complex algorithms to detect a deformation. Yuan et al. [26] and Donlon et al. [27] estimated the contact force and shear force by using a convolutional neural network from RGB lights and marker images. However, the sensor needs a large space for use in an MIS environment. Gessert et al. [28] proposed 4D deep learning with streams of volumes for OCT-based force estimation. A temporal history improves force estimation performance and predicts short-term force. Hwang et al. [15] inferred the interaction forces using only visual information in the case of three objects made from different materials. Shin et al. [29] proposed a sequential image-based attention network. The network estimated the force without using a haptic sensor. Lee et al. [30] proposed a convolutional neural network (CNN) and LSTM-based interaction force estimation method that applied the camera information and an electrical current as inputs. The addition of an electrical current as an input helped estimate the forces related to rigid objects. Marban et al. [7] proposed an recurrent convolutional neural network (RCNN) to estimate the interaction forces during robotic surgery. Six-axis force on the skin was predicted using images, the tool-tip trajectory, and grasper status. The RCNN consisted of a VGG [31] network to represent spatial information from the images, such as images of the deformation of the skin, and an LSTM network for temporal information. Despite numerous studies on substituting a force sensor with an image sensor, the occlusion, optical noise, and motion of the camera continue to create an incorrect force estimation from an image. To overcome the lack of image information for estimating the interaction force, the electrical current of the robot, or the robot position, was recently added to an image as an input of a neural network for estimating the interaction force [30].

The main contribution of this paper is a vision-based suture tensile force estimation method that relies on monocular images. Unlike previous methods that estimate a 2D or 3D deformation and then predict the interaction forces using the deformation, our proposed method considers the spatial and temporal information together through deep learning based on images and tool positions without applying deformation measurement algorithms. Furthermore, the test are occurred at the untrained view to verify its feasibility.

### 2.2. Inception-Resnet V2

It is essential to create feature vectors in deep learning based on an image. These feature vectors are compressed features and information contained in images. Commonly used deep-learning encoders applied to extract a feature vector include AlexNet [32], VGG [31] and Inception [33]. Among them, Inception-Resnet V2 [34] a widely used encoder network for image classification and achieves an excellent performance. Inception-Resnet V2 uses a combination of a residual connection and Inception V4 structures. The modules of the network have a multi-branch structure. In each branch, there are filters of various dimensions, such as 1 × 1, 3 × 3, and 5 × 5. In addition, each branch is combined through a concatenation. Inception modules with a split-transform-merging architecture have a representational ability [35]. Residual connections increase the effectiveness of the learning, making it possible to learn quickly. For the interior of the Inception-Resnet V2 network, 10× Inception-Resnet-B, 5× Inception-Resnet-C, Reduction-A, Reduction-B and Reduction-C modules are used [34].

## 3. Materials and Methods

In this section, a method is described for estimating the interaction force during suturing using images and tool positions to generate pseudo-tactile feedback, which has been investigated for its usefulness during teleoperation surgery [36]. Similar to experienced surgeons merging visual information and proprioception to feel a pseudo-tactile sensation, our method finds that the tension increases in the image even with slight changes at the end of the stitch as a result of the movement toward the direction of the knot pulling. To estimate the interaction force during the suturing motion during robotic surgery, the database was generated while a teleoperated robot was carrying out a suturing motion under master control. The dataset was entered into the network after augmentation and preprocessing.

### 3.1. Overall Operating System

**Figure 2:**
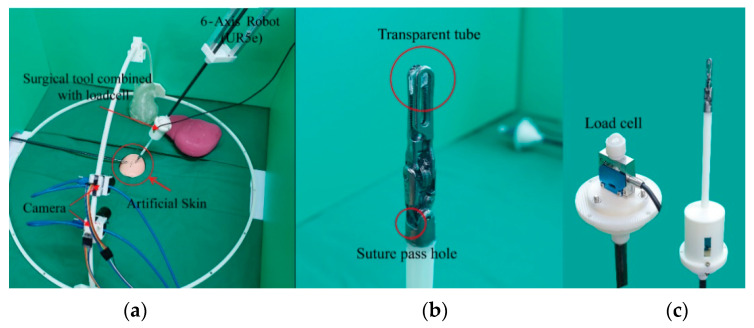
(**a**): Experimental setup. (**b**): Tool tip for measuring tensile force. (**c**): Instrument combined with load cell.

In this experiment, the surgical robot was controlled using a master device (Geomagic Touch, 3D SYSTEMS, Rock Hill, SC, USA). The surgical robot is a combination of a surgical instrument (DaVinci Xi ProGrasp™ Forceps, Intuitive, Sunnyvale, CA, USA) and a 6-axis robot (UR5e, Universal Robots, Odense, Denmark), as shown in Figure 2a. The haptic device moved the UR5e through position control using only visual information without position or force feedback. The suturing action of a surgical robot using a silk 5-0 material is recorded by two cameras (CM3-U3-131Y3C-CS Chameleon3, FLIR Systems, Wilsonville, OR, USA) to capture sufficient visual data. At the same time, the ground-truth tensile force of the suture is measured through a load cell (DBSM-3 LOADCELL, BONGSHIN LOADCELL, Gyeonggi-do, Korea) incorporated into the surgical instrument. As shown in Figure 2b,c, the suture passes through a transparent tube and transmits tensile force to the load cell. The transparent tube allows the forceps to pull without holding the thread. In this way, the tension on the suture can be measured from any position.

The position of the surgical tool was obtained from the controller of the 6-axis robot. Image data, tool position and ground-truth tensile force were obtained at 60 Hz based on the frame rate at which the DaVinci (Intuitive, Sunnyvale, CA, USA) surgical robot system operates [37].

### 3.2. Dataset

**Figure 3:**
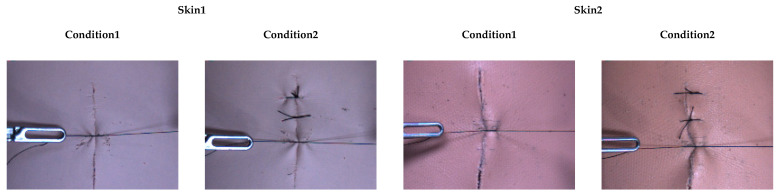
Two soft objects and two conditions per soft object used for experiment. Two artificial skin types with different elastic modulus: the elastic modulus of Skin1 is 387.23 kPa, and the elastic modulus of Skin2 is 68.26 kPa. Each artificial skin has two different conditions. Condition1 represents the start of the suture process. Condition2 represents an intermediate stage during the suture process.

From this environment, a database was built by using the camera views and target tensile force, where the suture interacts with two soft objects. Two soft objects were used with different elastic moduli. Each piece of artificial skin has a different elastic modulus, the elastic modulus of Skin1 is 387.23 kPa (which is similar to the epidermis), and the elastic modulus of Skin 2 is 68.26 kPa (which is similar to the dermis) [38]. Each soft object has two defined conditions: Condition1 represents the start of the suture process. Condition2 represents being at an intermediate stage during the suture process, as shown in Figure 3. The camera views can be changed by moving the camera itself along a semi-circular camera rail, as shown in Figure 2a. Images were recorded at 75°, 60° and 45° angles along the semi-circular camera rail for the training dataset, whereas images taken from 90°, 85°, 80°, 70°, 65°, 55°, 50°, 40°, 35° and 30° angles were used in the test dataset. After a target tensile force is set, the tensile force can be increased up to the target tensile force through the master device. The experimental time is determined by how long it takes for the target tensile force to be reached. The target tensile forces used for training data are 0.5, 1.0, 1.5, 2.0, 2.5 and 3.0 N. The target tensile forces used for testing are 1.0, 2.0 and 3.0 N. The baseline tensile force value for sutures, under both in vivo and in vitro conditions, is 2.37 ± 0.33 N for the silk 5–0 material [39]. For this reason, silk 5–0 sutures is used and chose a maximum target tensile force of 3.0 N to study conditions under which the baseline tensile force was exceeded.

The training dataset consisted of 288 combinations of data from two soft objects, two conditions, four applications for each of three camera views, and six target tensile force changes (2 soft objects × 2 conditions × (4 × 3) views × 6 target tensile forces). For the test data, a total of 120 combinations of data from 2 soft objects, 2 conditions, 10 camera view angles, and 3 changes in target tensile force were used (2 soft object × 2 conditions × 10 view angles × 3 target tensile forces). The experimental time for a single dataset is between 5 and 10 s. The dataset is a collection of data units, which consists of images, the synchronized ground-truth tensile force, and the position of the surgical tool at a given time. Finally, the 288 datasets in the training data are made up of 138,595 (69.53%) data units, and the 120 datasets in the test data are made up of 60,792 (30.47%) data units.

### 3.3. Image Augmentation and Data Preprocessing

Data augmentation is a training method that uses similar but different examples for training data [40]. Augmentation is a way to supply additional training pairs to simultaneously suppress an overfitting and improve the performance. Random cropping, random brightness, random saturation, random hue, and random contrast were used in our images as augmentations. For the random cropping, the location and size of the bounding box are randomly set over the image at between 0.5 and 1.0, restricting the ratio of width to height from 0.5 to 1.5. The delta for a random brightness is 0.125, the lower bound for the monitoring factor of a random saturation is 0.5, and the upper bound is 1.5. The maximal delta value of the random hue is 0.2, and the lower bound and upper bound of the random contrast factor are 0.5 and 1.5, respectively. In the preprocessing process, data are manipulated to allow the deep learning network to be effectively trained. Both the image and tool position data were preprocessed to facilitate learning. Image preprocessing changes the image pixel values from integers of between zero and 255 to float values of between zero and 1, subtracts 0.5 from all pixels, and multiplies by 2 to normalize the value of all pixels to a value between −1 and 1. Then, a resize method, which adapts binary interpolation, was used to create 320 × 240 pixel images from 640 × 480 pixel images. In addition, the position of the tool, the three-dimensional distance between the reference frame for the robot, and the reference frame of the tool, was normalized to a value within the range of zero to 1 by dividing the maximum moving distance of our experimental setup.

### 3.4. Feature Modeling Using Proposed Network

Because surgeons undertaking robotic surgery use both visual information and proprioception to predict interaction forces, a network architecture using images and the tool position together is used to estimate the interaction force during a suturing motion. Our method consists of two stages: spatial feature modeling and temporary feature modeling. Spatial feature modeling is an encoding network that expresses the features of the images. Serially connected temporal modeling is a network that predicts interaction forces using temporal information such as changes in the image features and the tool position.

#### 3.4.1. Spatial Feature Modeling

**Figure 4:**
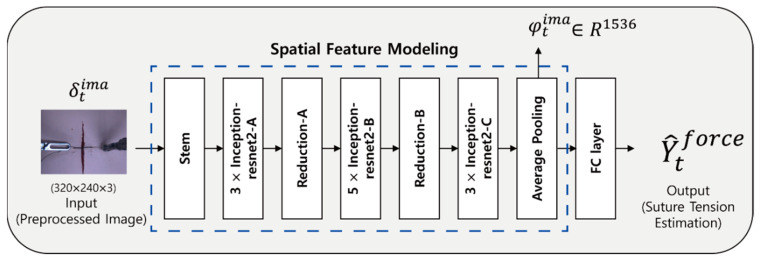
Spatial Feature Modeling structure. Inception-ResnetV2 network is used to obtain the spatial feature vector. This network consists of Stem, 3× Inception-resnet2-A, Reduction-A, 5× Inception-resnet2-B, Reduction-B, 3× Inception-resnet2-C, average pooling and FC layer modules. At test time, feature vector $\varphi_{t}^{ima}$ is extracted from the average pooling.

A lightweight Inception-resnetV2 network was used to improve the utility during actual use. As shown in Figure 4, the light-weighted Inception-resnetV2 network consists of 3 Inception-Resnet-A, 5 Inception-Resnet-B, a 3 Inception-Resnet-C blocks, a Stem module with a half filter size, and a reduction module with a half filter size compared to the original Inception-resnetV2. In this way, the parameter size was reduced by 40.4% from 55,096,033 of the original Inception-resnetV2 to 22,294,560 with the modified Inception-resnetV2. The modified Inception-resnetV2 will help provide real-time force feedback by reducing the calculation time in the force estimations. During the training stage, the softmax part of the original Inception-resnetV2 is replaced by FC layers to extract the spatial features from the images. The input for this process is a preprocessed image, and the output is an estimated tensile force. The output from this process is compared to the ground-truth tensile force synchronized with the image. This process causes a loss of the optimization, and the network is trained by this loss. The learning rate was 0.0001, and the batch size was 10. The network was trained using $L_{2}$ loss and an Adam optimizer [41] with $\beta_{1}=0.9,\;\beta_{2}=0.999$. By contrast, the test stage uses the feature vector of R^1536^, which is the output from the average pooling layer. The spatial feature modeling is trained separately before temporal feature modeling.

#### 3.4.2. Temporal Feature Modeling

**Figure 5:**
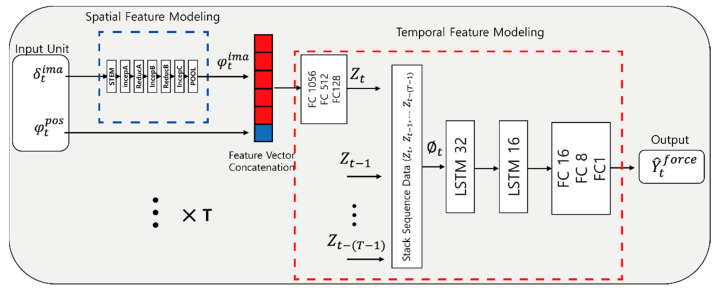
Proposed network structure. The network consists of spatial feature modeling and temporal feature modeling. Whereas spatial feature modeling and temporal feature modeling are trained separately, the two models trained are used together for the suture tensile force estimation during the test stage. Preprocessed images and preprocessed tool positions are used as input for the suture tensile force estimation. $\delta_{t}^{ima}\in R^{320\times 240\times 3}$ = preprocessed image, $\varphi_{t}^{ima}\in R^{1536}$ = feature vectors from the image, $\varphi_{t}^{pos}\in R^{3}$ = preprocessed tool position, and $Z_{t}\in R^{128}$ = FC output feature of the feature vector concatenation. The feature vector concatenation is a concatenation of the image feature and tool position, $\emptyset_{t}\in R^{T\times 128}$ = stacked sequence feature vector, ${\hat{Y}}_{t}^{force}$ $\in R^{1}$ = estimated suture tensile force, and T = sequence size.

As shown in Figure 5, the image feature vector R^1536^ derived from the average pooling layer of the learned spatial feature modeling and the position of the tool measured by the robot are concatenated. From the concatenated values, the outputs obtained through FC layers of 1056, 512, and 128 are regarded as the *Z*-values. By stacking the data, the *Z*-values change to stack sequence data according to the number of choices. The network is consist of 32 sequences of data in the LSTM layer with 32 and 16 nodes, and finally passed the data through the FC layer to estimate the suture tension. The purpose of this temporal feature modeling is to improve the tension estimation performance by using the changes in features over time.

## 4. Results

### 4.1. Results for Soft Objects and Conditions

**Figure 6:**
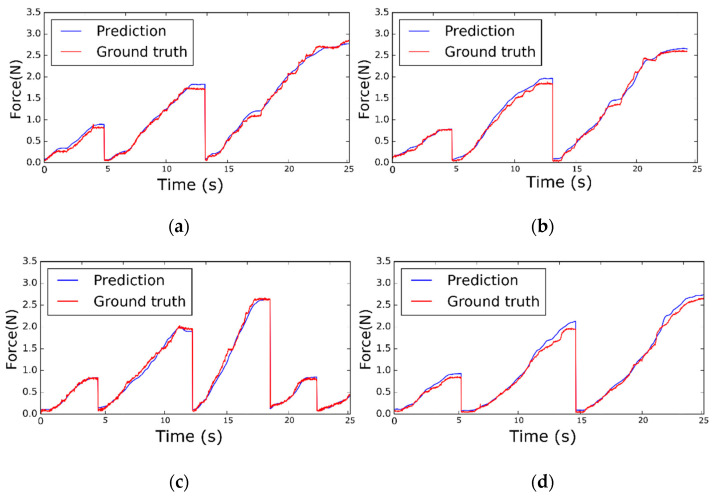
The estimated tensile force estimation results from the proposed network for two soft objects and two conditions: (**a**) Graph from 85° view for Skin1, Condition1, (**b**) graph from 70° view for Skin1, Condition 2, (**c**) graph from 55° view for Skin2, Condition1, and (**d**) graph from 40° view for Skin2, Condition 2. Sixty time steps are equal to 1 s.

In this section, the results of the proposed network learned through the collected datasets are presented, and its performance is analyzed. The estimated forces of the figures were calculated after data acquisition. In this study, the proposed network is trained on a PC with the assistance of a TITAN V GPU (NVIDIA, California, CA, USA). During the test, the calculation time used to predict the interaction force with an image is 12.13 ms, which fully proves that the design of the system meets the real-time requirements. The differences between the estimated force from the proposed network model and the measured ground-truth force was analyzed with quantified errors using the root mean square error (RMSE) and maximum absolute error (Max-AE) when the maximum pulling force was 3.114 N within the test set. Figure 6 shows a graph of the sample results of the ground-truth tensile force and estimated tensile force from 85°, 70°, 55°, and 40° untrained views for two soft objects and two conditions. Each soft object has two conditions, as shown in Figure 3. In both the early phase of a stitch, where the visual changes are in proportion to the tensile force (e.g., during the starting part of a knot), and in the latter phase of a stitch where there is a slight image change (e.g., finishing a knot), the tensile force of the sutures is well predicted.

Table 1 shows the RMSE and Max-AE between the ground-truth and estimated tensile [42,43]. The proposed model performs better for Skin 2 than Skin 1 under the same conditions to compare rows 1 and 3 as well as 2 and 4 of Table 1. Under the same conditions, Skin 2 showed more deformation for the same tensile force compared to Skin 1 because of its lower elastic modulus. In addition, based on Table 1, the results show that the proposed method has better results for condition 1 in terms of RMSE and Max-AE because the images under Condition1 are clearer in terms of deformation, given the same skin, than under Condition 2. Consequently, this means that the actual suture tensile force for soft objects is easier to estimate owing to the more apparent deformation.

### 4.2. Comparison of Results by Networks

**Figure 7:**
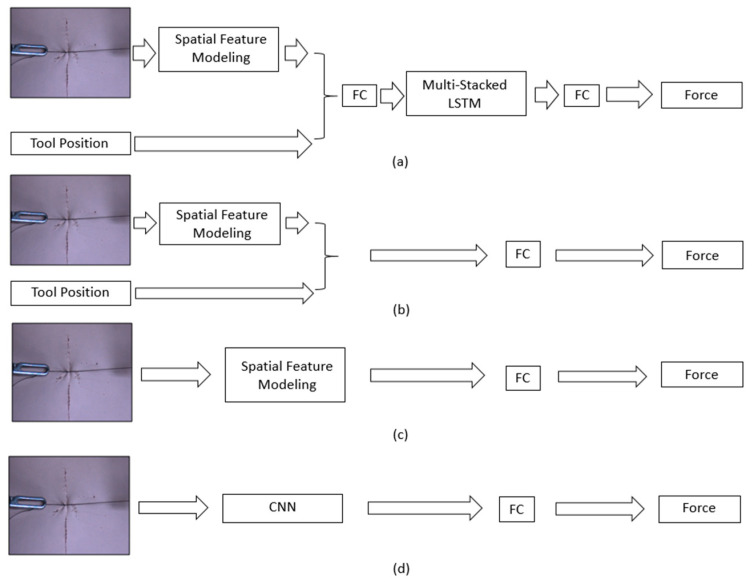
Network structure diagram: (**a**) proposed network, (**b**) comparison network 1 with inception-based spatial feature modeling and tool position, (**c**) comparison network 2 with Inception-based spatial feature modeling, and (**d**) comparison network 3 with CNN-based spatial feature modeling.

**Figure 8:**
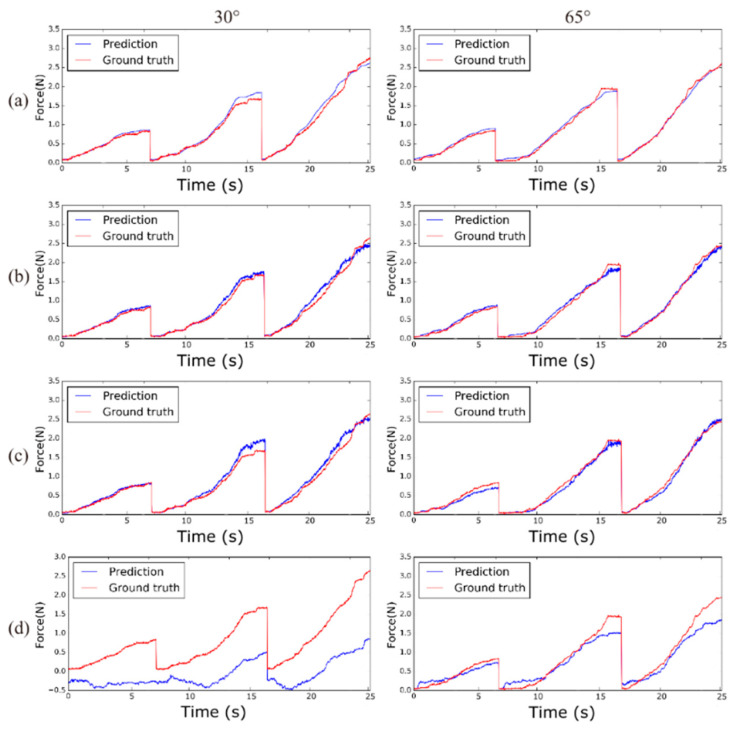
Tensile force estimation results from various networks. The estimation result for a 30° view is shown in the left column and the estimation result for a 65° view is shown in the right column: (**a**) proposed network, (**b**) comparison network 1, (**c**) comparison network 2, and (**d**) comparison network 3.

To verify the feasibility of the proposed method using images, tool position, and LSTM, our results were compared with comparison networks that use the image and tool position without an LSTM (comparison network 1), only an image with inception-based spatial feature modeling (comparison network 2), and only an image with CNN-based spatial feature modeling (comparison network 3), as shown in Figure 7. The comparison methods were trained using the same training data. Figure 8 shows a comparison graph of a sample of the ground-truth suture tensile force and estimated suture tensile force at 30° and 65°. In addition, Table 2 shows the results of the comparison cases in terms of RMSE and Max-AE at 90°, 65°, 55°, and 30°.

Table 2 and Figure 7 show that visual information can be used to estimate the suture tensile force. However, it is important to use the proper spatial feature modeling to encode an image. The comparison network 3, which uses a simple CNN encoder, shows significant errors in RMSE and Max-AE (RMSE = 0.4055, Max-AE = 1.6452). A reasonable performance was shown in comparison network 2, which uses inception-based spatial feature modeling (RMSE = 0.1005, Max-AE = 0.6662). In particular, there is a distinct difference in error between the comparison network 2 results and comparison network 3 results from 90° and 30° views, which is farther from the training views than the 65° and 55° views. Through these experiments, we showed that our proposed estimation method uses the image, tool position, and LSTM effectively. As a result, the proposed method shows the best performance for a suture tensile force estimation by presenting quantified errors.

## 5. Discussion

The experimental results in this study prove that our vision-based suture tensile force estimation method was able to estimate the suture tensile force for RAMIS systems. Our proposed method can estimate the suture tensile force in an environment where the view and type of soft object change. When suturing soft objects, the type of deformation varies depending on the elastic modulus of the soft object and the suturing condition, such as at the start or during an intermediate stage of the suture process. With the proposed method, a suture tensile force estimation was conducted for two soft objects under two conditions from various untrained views, verifying the feasibility of our method for RAMIS in a similar environment. In addition, our proposed method uses images and tool positions, as well as how such information changes in the same way as when an experienced surgeon uses visual information and proprioception together. Therefore, our method can improve the performance during the end part of a stitch where little deformation of the soft object occurs when the suture is pulled.

Figure 4 shows our proposed vision-based deep learning network. Our vision-based deep learning network uses images and tool positions as data input and consists of two parts: spatial and temporal feature modeling. The spatial feature modeling extracts image features from each image, and the temporal feature modeling identifies changes in the images and tool positions. As a result, our deep learning network can estimate the tensile force of sutures in soft objects under the conditions shown from untrained views. In previous studies, a force sensor was used to measure the interaction forces [8,9,10,11]; however, such methods suffer from numerous constraints such as cost, biocompatibility, sterilization, and miniaturization. Moreover, in previous studies using available information from models of the surgical tool and motor devices [13,14], the parameter values of the surgical tool need to be accurately known, and a precise accurate estimation is difficult because the motor is placed behind a trocar. To ease these constraints, previous methods used a vision-based force estimation with only visual information [15,29] or with both visual information and tool information [7]. In this study, our vision-based suture tensile force estimation method was more efficient as it used images and tool positions during the suturing action. Figure 8 shows that the use of images, tool positions, and the changes in this information give better suture tensile force estimation results than the other methods. Our vision-based deep learning network leverages the images and tool positions by combining different networks: inception-resnetV2 based spatial feature modeling for extracting image features and an LSTM-based temporal feature network for analyzing changes in the images and tool positions.

## 6. Conclusions

A suture tensile force estimation method based on monocular vision is proposed to apply to a surgical robot to provide interaction force feedback without force sensor. The proposed method estimates the tensile force through the proposed deep network based on modified Inception Resnet-V2 and LSTM networks using an image and tool position. The performance of the proposed method was verified with the generated DB, which recorded the image, robot position, and tensile force of the suture under the condition of two different artificial skins and two different situations (in vivo and in vitro) at 13 viewing angles of the images by changing the tool positions collected from the master-slave robotic system. The force estimation performance is compared by changing the inputs of the network by using spatial or temporal information. The results showed that the proposed method can estimate the suture tensile force in RAMIS systems with two types of artificial skin under the two conditions at various unseen views during training. As a further study, this vision-based force estimation method will be applied to a pig model used in open-heart surgery to estimate the interaction force in a complex environment. Moreover, the haptic feedback system or suture breakage alarm system in robotic surgery will be applied by using suture tensile force estimation method proposed in this study.

## Figures and Tables

**Table 1:** Results comparison for two objects and two conditions for untrained views.

Soft Object	Condition	RMSE (N)	Max-AE (N)
Skin1	1	0.07227	0.3286
Skin1	2	0.08065	0.4531
Skin2	1	0.07174	0.2479
Skin2	2	0.07847	0.2993
All test dataset		0.07578	0.4531

**Table 2:** Comparison of estimation method results in untrained views.

Model	Test Camera View Angle Accuracy					
		90°	65°	55°	30°	All Views
Proposed network	RMSE	0.0959	0.0627	0.0744	0.0983	0.0758
	Max-AE	0.3249	0.2110	0.256	0.4531	0.4531
Comparison network 1	RMSE	0.1011	0.0682	0.0766	0.1108	0.0816
	Max-AE	0.3995	0.2941	0.3244	0.4396	0.4396
Comparison network 2	RMSE	0.1348	0.0777	0.08729	0.1395	0.1005
	Max-AE	0.6662	0.3784	0.3567	0.5730	0.6662
Comparison network 3	RMSE	0.6458	0.2214	0.2617	0.6428	0.4055
	Max-AE	1.179	0.7819	0.7457	1.6452	1.6452

## Data Availability

Data sharing not applicable.
